# Double Puzzle: Morphogenesis of the Bi-Layered Leaf Adaxial Epidermis of *Magnolia grandiflora*

**DOI:** 10.3390/plants11243437

**Published:** 2022-12-09

**Authors:** Emmanuel Panteris, Ioannis-Dimosthenis S. Adamakis

**Affiliations:** 1 Department of Botany, School of Biology, Aristotle University of Thessaloniki, 54124 Thessaloniki, Greece; 2 Section of Botany, Department of Biology, National and Kapodistrian University of Athens, 15784 Athens, Greece

**Keywords:** multi-layered epidermis, wavy epidermal cells, morphogenesis, microtubules, cell wall

## Abstract

Anticlinal ordinary epidermal cell wall waviness is a widespread feature found in the leaves of a variety of land plant species. However, it has not yet been encountered in leaves with multiple epidermides. Surprisingly, in *Magnolia grandiflora* leaves, ordinary epidermal cells in both layers of the bi-layered adaxial epidermis exhibit wavy anticlinal contour. During the development of the above cells, cortical microtubules are organized in anticlinally oriented bundles under the anticlinal walls, and radial arrays extending from the bundles at the edges of anticlinal and external periclinal walls, under the external periclinal walls. This microtubule pattern is followed by cell wall reinforcement with local thickenings, the cellulose microfibrils of which are parallel to the underlying microtubules. This specialized microtubule organization and concomitant cell wall reinforcement is initiated in the external epidermal layer, while hypodermis follows. The waviness pattern of each epidermal layer is unrelated to that of the other. The above findings are discussed in terms of morphogenetic mechanism induction and any implications in the functional significance of ordinary epidermal cell waviness.

## 1. Introduction

During the conquest of terrestrial habitats, vascular plants “invented” an intriguing feature in leaf epidermis: ordinary epidermal cells with wavy anticlinal contour, which are found as early as in ferns of the Palaeozoic [1]. Throughout evolution, this feature became widespread among numerous—but not all—plant species belonging to all major vascular plant groups, exhibiting several morphological variations [2,3,4,5]. Starting from almost rectangular or polygonal protodermal cells, ordinary epidermal cells grow in a highly co-ordinated pattern: differential growth at specific areas of epidermal cells results in the shaping of “jigsaw-puzzle” cell layers, the adaxial and abaxial leaf epidermides [6,7].

The morphogenetic mechanism of the above ordinary epidermal cells has been studied thoroughly, revealing the roles of the cytoskeleton, the cell wall and a well-equipped toolbox of regulatory molecular switches [6,8,9,10,11,12,13]. Moreover, research in the field still flourishes, adding new components to the network of factors that control epidermal cell morphogenesis, such as FERONIA kinase [14,15], brassinosteroids and ROPGAP regulators [16,17], and MAPs [18]. Although this research field has existed for more than 80 years (Watson 1942 [19] and the literature cited therein), it keeps its timely vigor, recruiting more and more researchers.

Apart from the mechanism that is responsible for wavy epidermal cell contour, an intriguing issue is the advantage that such a shape may offer [5]. The functional importance of “jigsaw-puzzle” structure has been related to the unique location of leaf epidermis and the challenges that are imposed on this tissue. Being the border between the leaf and the air, leaf epidermis must be integral, flexible and well-adapted to any stresses applied by the underlying tissues and/or the environment. Wavy anticlinal wall shaping may offer stress relaxing during tissue development [20] and/or provide elasticity [21] against stretching and bending due to environmental forces [22,23]. 

Several plant species have been used as material in studies of wavy epidermal cell morphogenesis, including ferns, dicots and monocots, in which the same mechanism functions, although with variability in the fine details [4]. Of course, *Arabidopsis thaliana* has become the material of choice for the majority of recent studies (see reviews above and original articles published in 2022). Taken as implied, a common feature of all plant species studied for wavy epidermal cell morphogenesis is that their leaves have single-layered epidermides, while in leaves with multiple epidermides the anticlinal walls of ordinary epidermal cells are straight. In fact, observations in dicot (*Ficus elastica*) and monocot (*Tradescantia pallida*) species with multiple leaf epidermides (our unpublished data) confirm this consensus. 

Multiple epidermis, present in the leaves of several plant genera, including *Piper* [24], *Camelia* [25], *Peperomia* [26], members of the Cyperaceae [27], etc., is considered as an enhanced xeromorphic and scleromorphic feature [28] or function as a “window” focusing light to the photosynthetic tissues [29]. “Genuine” multiple epidermides derive from periclinal divisions of protodermal cells, while when periclinal divisions of leaf ground meristem provide the inner cell layers, the latter are characterized as a “hypodermis” [30]. In both cases, regardless of their ontogenesis, the absence of typical chloroplasts is a reliable criterion to recognize the layers of multiple epidermides from the underlying mesophyll [24]. Apparently, given their rigidity and solid structure, multiple epidermides could be considered as fortified enough, so that wavy anticlinal walls would not offer any further advantage. Nevertheless, *Magnolia grandiflora* is an exception, with a bi-layered adaxial leaf epidermis consisting of cells with wavy anticlinal walls. Accordingly, in this study we investigated the morphogenesis of this peculiar leaf epidermis in terms of microtubule organization and cell wall reinforcement. In particular, we focused on the relation among the two cell layers in the course of their development and the putative advantage of wavy anticlinal cell walls in a bi-layered epidermis.

## 2. Results

**Figure 1:**
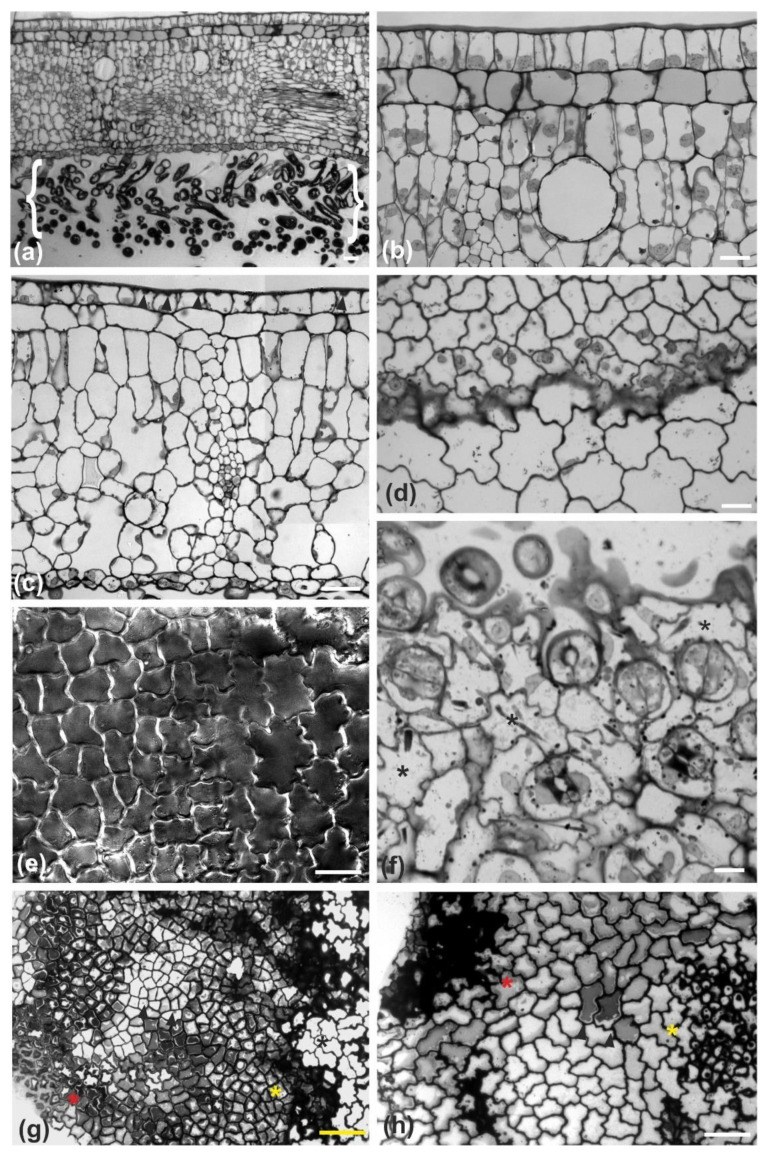
Light micrographs of developing (**a**–**f**) and fully mature (**g**,**h**) adaxial (**a**–**e**,**g**,**h**) and abaxial (**f**) leaf epidermis of *Magnolia grandiflora*, after toluidine blue staining of transverse (**a**–**c**) and paradermal (**d**, **f**–**h**) sections, or hand-cut section viewed with DIC optics (**e**). (**a**) Bi-layered adaxial and single-layered abaxial epidermis can be observed in this young leaf, the latter covered by a thick layer of trichomes (brackets). At higher magnification, it is observed that cell profiles of the two adaxial epidermal cell layers are totally unrelated (**b**). At a later developmental stage (**c**, see local wall thickenings pointed by arrowheads in the external epidermal cells), both adaxial epidermal layers appear continuous and integral. The cells of the external layer are smaller than those of the hypodermis, as observed at transverse (**c**) and paradermal (**d**) view. This was also confirmed by DIC observations of fresh unfixed epidermis (**e**); the external epidermis is on the left side of the image and the hypodermis on the right. Additionally, note that the waviness pattern among the two epidermal layers is totally unrelated (**d**,**e**). (**f**) Stomata are restricted to the abaxial epidermis, the ordinary epidermal cells of which (asterisks) exhibit wavy contour at paradermal view. (**g**) Top view of mature external epidermis, revealing that the waviness of external epidermal cells is more pronounced at their outer part (red asterisk) and close (yellow asterisk) to the hypodermis (black asterisk), while it appears diminished at their mid-height (cells pointed to by arrowheads). Similarly, the cells of the hypodermis (**h**) exhibit maximum waviness (red asterisk) just beneath the external epidermis and close to the mesophyll (yellow asterisk), while less waviness is observed at the mid-height of hypodermal cells (cells pointed to by arrowheads). Scale bars: 20 μm.

**Figure 2:**
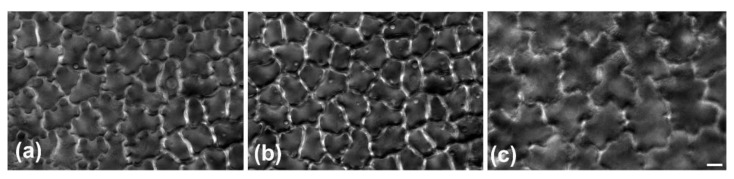
DIC micrographs of an adaxial epidermis area at paradermal view. (**a**,**b**): External epidermal layer at surface (**a**) and median (**b**) plane of focus. Note the diminishing of waviness in (**b**). (**c**) Hypodermis just beneath the cells depicted in (**a**,**b**); hypodermal cells are larger than the overlying epidermal ones. Their shape is not related to that of the overlying epidermal cells. Scale bar: 10 μm.

**Figure 3:**
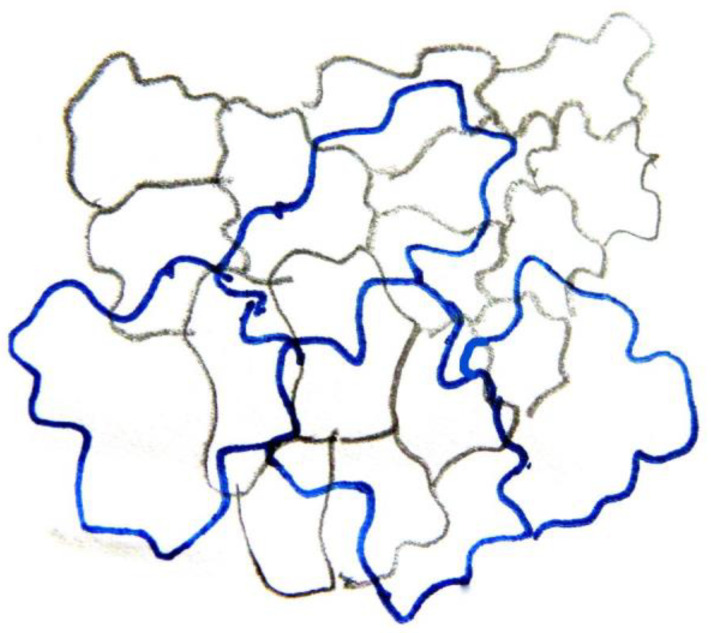
Camera lucida drawing of the two layers of adaxial leaf epidermis at paradermal view. External epidermal cell contours appear in black and hypodermal cell contours in blue. The cell contour of each layer is unrelated to that of the other.

The leaf of *Magnolia grandiflora* has a bi-layered adaxial epidermis and a single-layered abaxial one (Figure 1a). As observed by transmission electron microscopy (TEM), the two outer adaxial cell layers do not contain chloroplasts but a particular plastid type, with globular electron-dense inclusions (Figure S1a). The leaves are strictly hypostomatic, while the abaxial epidermis is covered by a thick “carpet” of trichomes (Figure 1a). Apparently, the adaxial epidermis consists of an external epidermal layer and a hypodermis, which, at cross section, do not exhibit any cell wall continuity at both early stages of leaf development (Figure 1b) and after completion of growth (Figure 1c). In addition, cell contours of each layer do not overlap or match those of the other at surface view (Figure 2 and Figure 3). Furthermore, observations of very young leaves revealed the occurrence of subepidermal periclinal divisions in the ground leaf meristem (Figure S1b). The cells of the external adaxial epidermal layer are smaller than those of the hypodermis (Figure 1d,e, Figure 2 and Figure 3). 

As they grow, all ordinary epidermal cells of both adaxial and abaxial epidermides exhibit wavy anticlinal contour (Figure 1d–f). Importantly, the waviness patterns, among the two layers of the adaxial epidermis, appear totally unrelated (Figure 1d,e, Figure 2 and Figure 3). However, a common feature among the adaxial epidermal layers is that waviness is not uniform throughout the height of anticlinal walls: maximum waviness occurs close to the periclinal walls, while it is less pronounced at the mid-height of each cell layer (Figure 1g,h).

**Figure 4:**
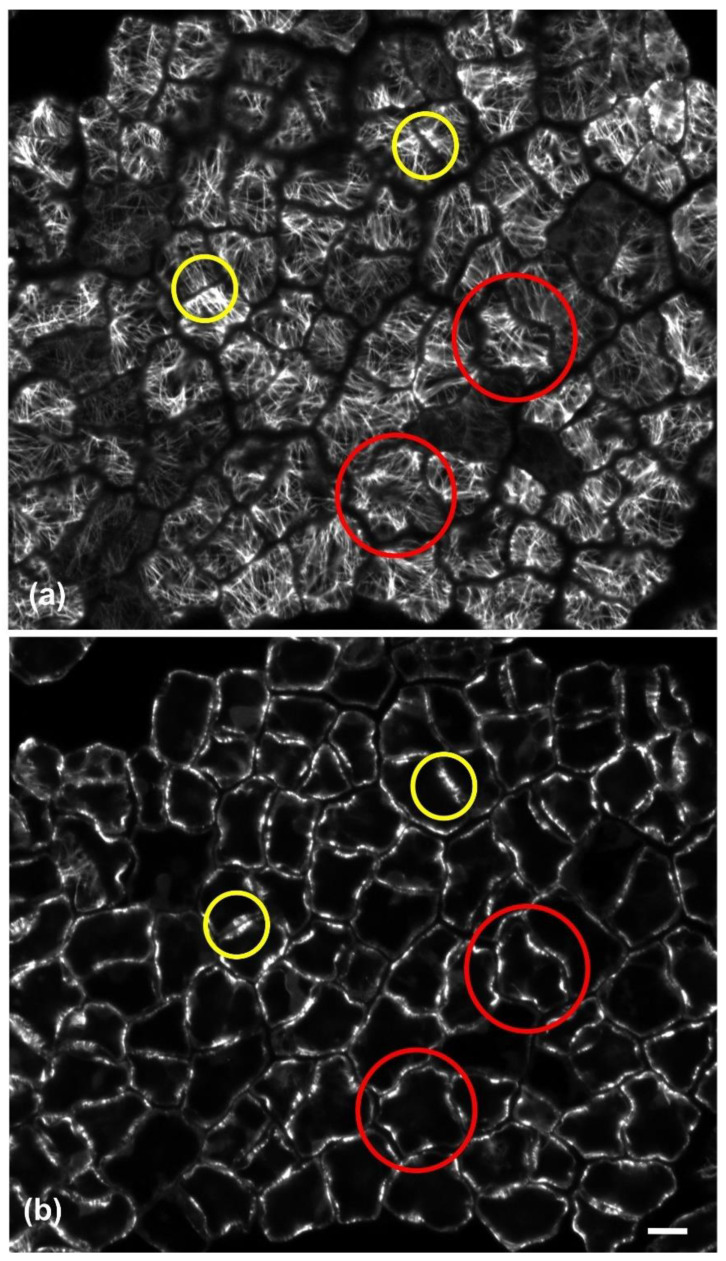
Single CLSM sections depicting microtubule organization in developing cells of the external adaxial leaf epidermal layer after tubulin immunostaining. Radial microtubule arrays extending under the external periclinal wall (**a**) and microtubule bundle profiles at median anticlinal wall height (**b**) can be observed at the same epidermal area. Microtubule arrays with alternating arrangement among adjacent cells are included in red circles, while those with opposite arrangement are included in yellow circles. Scale bars: 10 μm.

**Figure 5:**
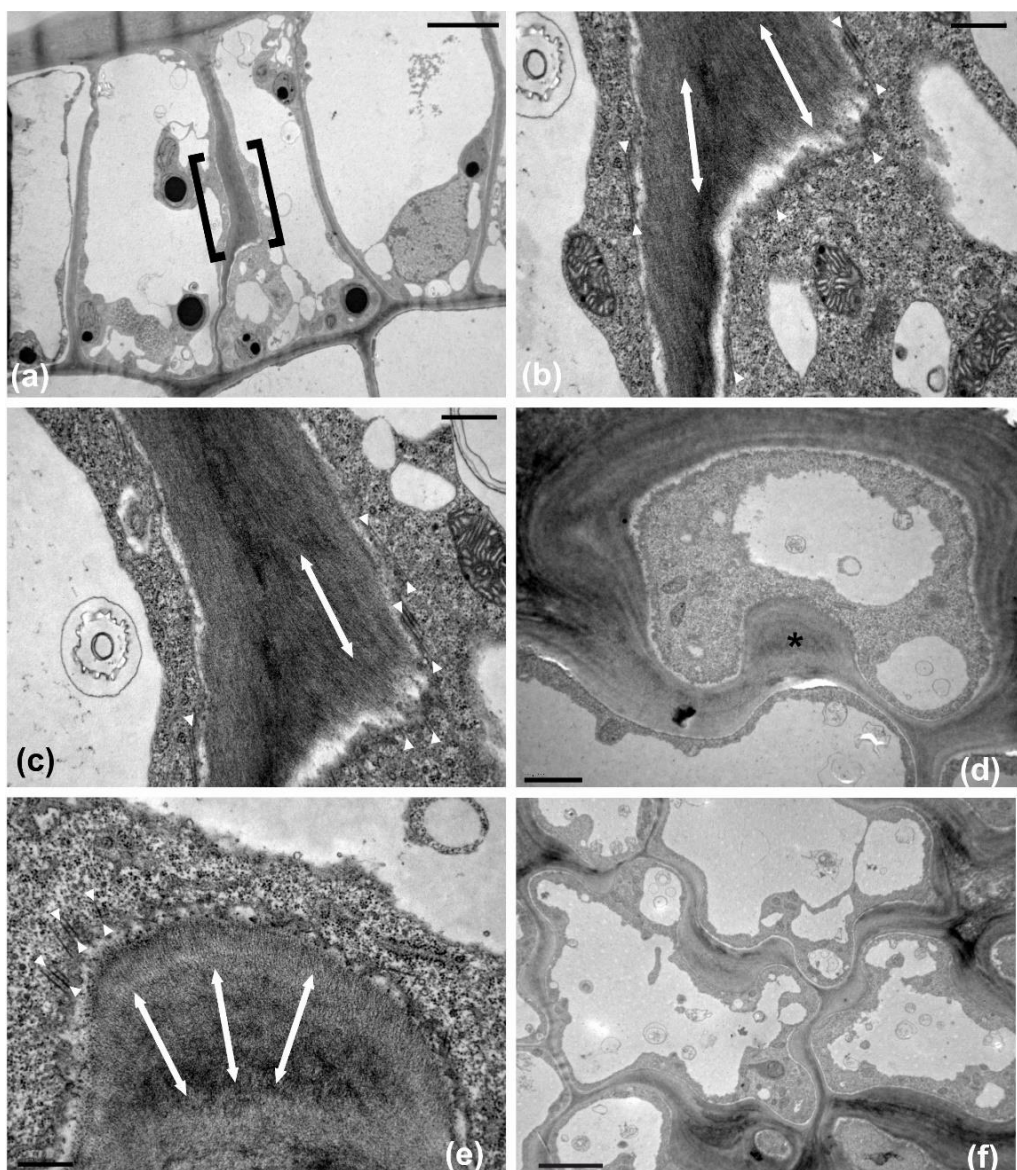
TEM micrographs of transverse (**a**–**c**) and paradermal (**d**–**f**) sections of external epidermal cells of the adaxial leaf epidermis. (**a**–**c**) Grazing section through an anticlinal wall thickening (within brackets in **a**). The wall is thickened in the cell at the right. At higher magnification (**b**,**c**), cellulose microfibrils within the thickening exhibit uniform anticlinal orientation (double arrows). Cortical microtubules that line the thickening (arrowheads in **b**,**c**) are strictly anticlinally oriented parallel to the cellulose microfibrils. Additionally, note that microtubules at the unthickened wall side are anticlinal as well (arrowheads in left part of **b**). (**d**,**e**) Cell wall pad (asterisk in **d**) at the junction of the external periclinal and anticlinal walls. At higher magnification (**e**), cortical microtubules that line the pad (arrowheads), as well as cellulose microfibril within the pad (double arrows), exhibit radial arrangement. (**f**) Local cell wall thickenings are alternating among adjacent cells. Scale bars a, d: 2 μm, b, c, e: 0.5 μm, f: 5 μm.

Observations of immunofluorescent samples by confocal laser scanning microscopy (CLSM) and of samples fixed and sectioned for TEM revealed that, in both layers of the adaxial epidermis, anticlinal waviness was the outcome of the same morphogenetic mechanism already found in various plant species (see Introduction). During development, cortical microtubules under the anticlinal epidermal cell walls exhibited a uniform anticlinal orientation, observable by both CLSM (Figure 4b) and TEM (Figure 5b). These microtubules are organized in anticlinally oriented bundles (Figure 4b), which, at the edges of the external periclinal and anticlinal walls, extend as radial systems under the periclinal wall (Figure 4a). At the sites of the above cortical microtubule clusters, cell walls are reinforced by local thickenings, the cellulose microfibrils of which are parallel to the underlying microtubule systems. 

In particular, wall thickenings with anticlinally oriented cellulose microfibrils are deposited under the anticlinal microtubule bundles (Figure 5a–c), while cell wall “pads” with radially arranged cellulose microfibrils are deposited at the sites of radial microtubule systems (Figure 5d,e). In general, the above microtubule systems and the overlying cell wall thickenings exhibit an alternating distribution among adjacent cells of each tissue layer, which results in the formation of protrusions (lobes) and constrictions (necks): microtubules appeared aggregated at the necks, while they were scarce at the adjacent lobe tips (Table 1). This arrangement does not allow the formation of intercellular spaces among epidermal cells (Figure 5f). Each cell of the external epidermal layer usually formed one lobe and one neck (two bending sites) with each of its lateral neighbors, while more lobes and necks are formed between laterally adjacent cells of the hypodermis, probably due to their larger size (Table 2). As an exception to the above arrangement, microtubule clusters with opposite arrangement have been observed among certain adjacent ordinary epidermal cells of the external layer (Figure 4) but not of the hypodermis. Obviously, the same mechanism is responsible for the anticlinal waviness of ordinary epidermal cells in the abaxial epidermis (data not shown).

**Figure 6:**
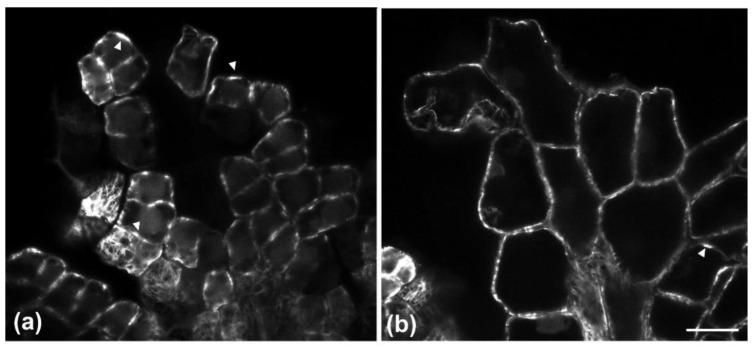
Single CLSM sections depicting microtubule organization in early developing cells of the external epidermal (**a**) and hypodermal (**b**) adaxial layers, at the same leaf area, after tubulin immunostaining. Microtubule bundles are already organized in external epidermal cells (arrowheads in **a**), while microtubule bundle initiation is just beginning in some cells of the hypodermis (arrowhead in **b**). Scale bar: 20 μm.

Initiation of the above mechanism is not synchronized in the two layers of the adaxial epidermis. It was observed that while cortical microtubules were already organized in bundles in the external layer of the adaxial epidermis (Figure 6a), formation of bundles was just initiating in the underlying cells of the hypodermis (Figure 6b). The opposite, i.e., the occurrence of microtubule bundles in cells of the hypodermis but not in those of the covering external layer of epidermis, was never observed.

**Figure 7:**
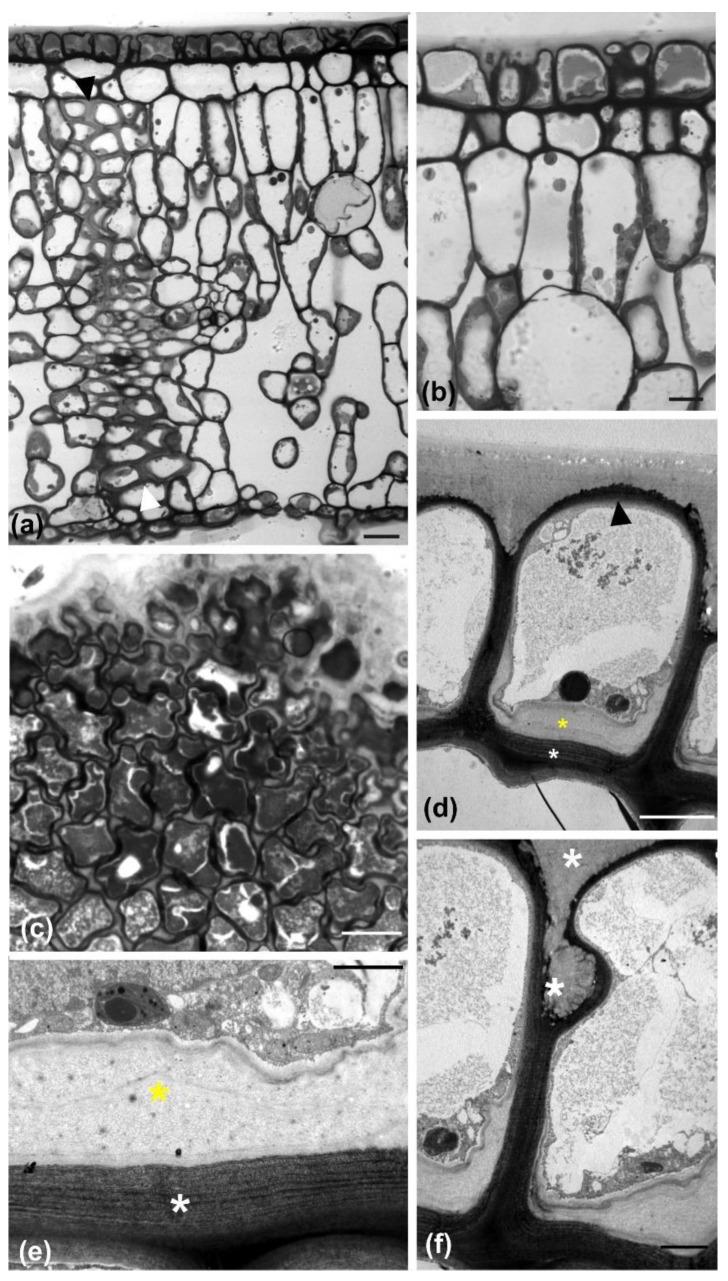
Light (**a**–**c**) and TEM (**d**–**f**) micrographs of mature leaves at transverse (**a**,**b**,**d**–**f**) or paradermal (**c**) section. At low magnification of transverse section (**a**), a “wall” of sclerenchyma cells can be observed (arrowheads) extending between the adaxial and abaxial epidermides. In addition, the external adaxial epidermal cells appear partially detached, which is shown at higher magnification in (**b**). The detachment is also visible at grazing paradermal section (**c**). (**d**) At TEM level, external adaxial epidermal cells exhibit a polylamellate inner periclinal wall (white asterisk), thicker than the external periclinal one (arrowhead), lined by the deposition of polysaccharide material (yellow asterisk). (**e**) Higher magnification of an inner periclinal wall area, like that in (**d**), with same labeling. (**f**) Higher magnification of a detachment site between two adjacent adaxial epidermal cells, depicting the stuffing with cuticle material (asterisks). Scale bars a: 20 μm, b, d: 5 μm, c: 10 μm e, f: 2 μm.

During their development, the external adaxial epidermal cells are closely packed, forming an integral layer throughout the whole height of their anticlinal walls. As a result, the external periclinal walls resemble a continuous “roof” covered by the cuticle (Figure 1a–c). At full maturity, however, the external adaxial epidermal cells appear partially detached and deformed, almost to the upper half of their anticlinal height, while the gaps between them are sealed with thick cuticle deposition (Figure 7). Obviously, this conformation and cuticle thickening establishes a strong hydrophobic shield over the adaxial leaf epidermis as a whole. Contrarily to the external adaxial epidermis, the hypodermis remains intact at maturity, without any gaps among its cells (Figure 1h and Figure 7a,b).

## 3. Discussion

It was surprising that cells of both adaxial epidermal layers of *M. grandiflora* leaves exhibited wavy anticlinal contour. As these layers appear ontogenetically and developmentally independent from each other, they may be considered as two distinct cell sheets, which, however, adopt the very same morphogenesis. Although the role of anticlinal cortical microtubules in local cell wall thickening has been disputed by real-time observations in *Arabidopsis thaliana* epidermis [31], their presence in bundles under the relevant wall thickenings in *M. grandiflora* was prominent. Accordingly, whether a specific cell wall area will become a “lobe” or a “neck” is determined by the local density of cortical microtubules: although uniformly anticlinally oriented, their population becomes the marking tool for the control of cell wall thickening and shaping (Table 1), in combination with the radial microtubule systems under the external periclinal wall, in both adaxial epidermal layers. Apart from the microtubule-driven mechanism, which has been extensively discussed previously (see Introduction), there are two major issues for interpretation. First, where might the above mechanism be initiated? Second, what might anticlinal wall waviness, which is confined horizontally, offer as an advantage to the bi-layered adaxial epidermis? Furthermore, what light does this wavy bi-layered epidermis shed on any reasonable advantage of wavy walls *vs.* straight walls in general?

Since morphogenesis starts at the external epidermal layer, followed by the hypodermis, it can be assumed that its induction does not derive from the underlying mesophyll tissue. This is in accordance with the case of plants, such as *Begonia lucerna*, in which leaves contain mesophyll cells but petals do not, consisting only of epidermides and venation. In such plants, while leaf epidermides are made of cells with straight walls, petal epidermal cells exhibit prominent waviness [4]. It appears that the case of *Adiantum capillus-veneris* leaflets, where epidermal cell waviness appears to be “imposed” by the underlying mesophyll [3], is an exception related to the anatomical peculiarity of this fern’s leaflet. Besides, in vitro-grown mesophyll-less leaflets of this fern exhibit ordinary epidermal cells, which follow the same morphogenetic pattern of most higher plant species, so mesophyll is not required to trigger wavy cell shaping [4]. It might thus be concluded that, wherever present, the “jigsaw-puzzle” pattern is an intrinsic feature of the epidermis, not related to the underlying tissues. Concerning the *M. grandiflora* hypodermis, it is tempting to consider the triggering of the same morphogenetic mechanism as “contagious”, transmitted from the external cell layer to the hypodermal one. In terms of time, this is supported by the sequence of events, as hypodermal cells always followed in microtubule clustering those of the external epidermis. In previous studies, it was supported that microtubule organization in ordinary epidermal cells is preceded by and probably due to mechanical stress imposed on them [32,33]. It is tempting, therefore, to suggest that differential local growth of the external adaxial epidermal layer, during morphogenesis, results in mechanical stress imposed on the underlying hypodermal cells, thus inducing microtubule organization in bundles and radial systems.

Among several authors, Jacques et al. (2014) [34] asked: “why shaping wavy cell contours?” Two views appear to be more convincing in answering the above question, concerning the mechanical and structural significance of epidermides with wavy anticlinal walls: it has been supported [20] that the wavy anticlinal wall contour of ordinary epidermal cells may serve as a “relaxing” mechanism, minimizing the mechanical stress created by growth events during epidermal development. The spreading of forces horizontally, as it appears to occur by creating the “puzzle” configuration, allows avoiding the outward bulging of external periclinal epidermal cell wall [20]. Alternatively, it has been shown that wavy anticlinal walls may act as “springs”, allowing a reversible change in mature epidermal cell shape, thus warranting the integrity of leaf epidermis under mechanical stresses due to environmental challenges [21]. Importantly, these two views do not exclude each other, as they clearly differ in terms of “timing”: the first [20] relates anticlinal waviness with pre-maturity stress during cell and tissue growth, while the second [21] implicates epidermal cell waviness with environmental mechanical stresses imposed after completion of growth. It is thus tempting to consider this morphogenetic mechanism as “killing two birds with one stone”.

However, the case of *M. grandiflora* leaf appears to challenge the “stress minimizing” view [20]. As the adaxial hypodermis is a cell layer “sandwiched” between the overlying external epidermal cells and the underlying mesophyll, its cells are confined all around. As a consequence, there is no free area for them to bulge outwards or inwards. Accordingly, the adaxial hypodermis may not experience the mechanical stresses that should require a special mechanism to be dealt with. 

On the other hand, the leaf of *M. grandiflora* is stiff but flexible. Its stiffness is partly due to its internal fortification by thick sclerenchymatic extensions form the vascular bundles towards both the adaxial and abaxial epidermides (Figure 7a). In addition to the above “endoskeleton”, the presence of a bi-layered and bi-wavy adaxial epidermis, combined with the also wavy abaxial epidermis, may offer the advantage of an “exoskeleton”, increasing the rigidity of the whole leaf structure. In fact, the structure of *M. grandiflora* leaf is highly reminiscent of that found in airplane wings, simultaneously flexible and rigid [35]. 

Furthermore, it could be assumed that the partial separation of the cells in the external adaxial epidermal layer may affect its rigidity. Although stuffing among cell gaps with thick cuticle transforms the external epidermal layer into a highly hydrophobic barrier, its overall continuity and integrity may diminish. Notably, opposite arrangement of microtubule bundles and radial arrays occurs among some adjacent epidermal cells in the external layer (Figure 4). Such an opposite arrangement has been considered as a feature of mesophyll cells, promoting the opening of intercellular spaces by cell separation, as observed in fixed [36,37] and living cells [38]. Therefore, it appears that the partial detachment among ordinary epidermal cells of the mature external layer is not just a “side-effect” of cuticle layer thickening, but it is also facilitated by a sophisticated hybrid morphogenetic mechanism. As a result, the integrity and elasticity of the whole adaxial epidermis relies on the hypodermal layer, the cells of which, apart from exhibiting wavy anticlinal contour, remain firmly attached to each other. In other words, the waviness of anticlinal walls in adaxial hypodermal cells may represent a “spare” machinery, in order to support the integrity and flexibility of the adaxial epidermis, which may weaken due to the partial detachment of the external epidermal layer cells.

## 4. Materials and Methods

### 4.1. Plant Material—Chemicals and Reagents

Young early sprouting leaves were collected from *Magnolia grandiflora* trees of a municipal garden in Thessaloniki from mid-April until mid-May. Mature fully grown leaves were collected in May. Both young and mature leaves were immediately prepared for light microscopy and TEM, as well as for immunofluorescence, for examination with CLSM. Leaf sampling was repeated 5 times, including at least 4 young and 4 mature leaves each time, for both TEM and CLSM. The chemicals and reagents used in sample preparation were purchased from Applichem (Darmstadt, Germany) and Sigma (Steinheim, Germany), unless otherwise stated. All preparation steps were performed at room temperature, unless specified otherwise.

### 4.2. Light Microscopy and TEM

For light microscopy and TEM, young and mature leaf laminas were cut with razor blades to pieces ~3 × 3 mm^2^ and fixed immediately with 3% (*v*/*v*) glutaraldehyde (Polysciences) in 50 mM sodium cacodylate buffer, pH 7, for 4 h. After 3 × 15 min rinses in the same buffer, the leaf pieces were post-fixed at 4 °C overnight with 1% (*w*/*v*) osmium tetroxide in the same buffer. After 3 × 15 min rinses in the same buffer, the leaf pieces were dehydrated in an acetone series, treated twice with propylenoxide and finally embedded in Spurr’s resin. Semithin sections (1.5–2 μm thickness) were cut with glass knives, stained with 1% (*w*/*v*) toluidine blue O in 1% (*w*/*v*) borax solution and observed under a Zeiss Axioplan (Carl Zeiss AG, Germany) or a Zeiss Axio.Imager Z2 (Carl Zeiss AG, Germany) light microscope. In addition, hand-cut paradermal sections of fresh unfixed leaves were observed with the DIC optics of the above microscopes. Digital micrographs were recorded with a Zeiss Axiocam MRc5 (Carl Zeiss AG, Germany), with Axio.Vision (Carl Zeiss AG, Germany) or ZEN 2 software (Carl Zeiss AG, Germany), following the instructions of the manufacturer.

Ultrathin (60–90 nm) sections were cut with a glass or diamond knife, collected on copper grids, double stained with 2% (*w*/*v*) uranyl acetate in 70% (*v*/*v*) ethanol solution and 1% (*w*/*v*) Reynolds’ lead citrate, and examined with a JEOL JEM 1011 TEM at 80 kV. TEM micrographs were recorded with a GATAN 500 digital camera, using the Digital Micrograph 3.11.2 software, according to the manufacturer’s instructions.

### 4.3. Tubulin Immunofluorescence—Confocal Microscopy

For microtubule immunostaining, young leaves were cut with razor blades into small (~2 × 2 mm^2^) pieces, which were immediately fixed for 1.5 h in 4% (*w*/*v*) paraformaldehyde + 5% (*v*/*v*) dimethylsulfoxide (DMSO) in PEM buffer (50 mM PIPES, 5 mM EGTA, 5 mM MgSO_4_, pH 6.8). After 3 × 15 min rinses with PEM, the leaf pieces were treated for 2 h with an enzyme cocktail (3% (*w*/*v*) cellulase R-10 (Duchefa) + 2% (*w*/*v*) macerozyme R-10 (Duchefa) in PEM) for cell wall digestion. Following rinsing with PEM, the pieces were treated with absolute frozen methanol at −20 °C for 30 min, then extracted with 5% (*v*/*v*) DMSO + 1% (*v*/*v*) Triton X-100 in PBS for 1 h, and afterwards incubated overnight with rat anti-*α*-tubulin (YOL1/34, Santa Cruz) diluted 1:40 in PBS. After 3 × 15 min rinses with PBS, the pieces were incubated overnight with AlexaFluor488-anti-rat (Cell Signalling) diluted 1:300 in PBS. After a final rinse, the leaf pieces were mounted on microscope slides in a drop of antifade solution (glycerol + PBS (2:1, *v*/*v*) + 0.5% (*w*/*v*) p-phenylenediamine). The above fluorescent specimens were examined with a Zeiss Observer.Z1 inverted microscope, equipped with LSM780 CLSM module and ZEN2011 software. Digital micrographs were recorded following the instructions of the manufacturer.

### 4.4. Measurements and Statistics

Microtubule number at lobe tips and necks was measured in a sample of 50 TEM micrographs. The abundance of cell lobes/necks among neighboring cells was measured by light microscopy in samples of external epidermal and hypodermal cells, comprising 50 cells each. Statistical differences were assessed with a *t*-test. The threshold for significance was set to *p* < 0.05. All analyses were carried out in GraphPad Prism (San Diego, CA, USA).

## Figures and Tables

**Table 1:** Microtubule number at protrusions (lobes) and constrictions (necks). Data presented are a mean ± standard error of 50 measurements.

Number of microtubules at cell necks	23.4 ± 0.64 *
Number of microtubules at lobe tips	3.5 ± 0.3 *

* *p* < 0.05.

**Table 2:** Number of lobes/necks among neighboring cells of the external layer and the hypodermis. Data presented are a mean ± standard error of 50 measurements.

Number of lobes/necks among neighboring cells external layer	1.9 ± 0.08 *
Number of lobes/necks among neighboring cells hypodermis	3.6 ± 0.1 *

* *p* < 0.05.

## Data Availability

Not applicable.

## Supplementary Materials

The following supporting information can be downloaded at: https://www.mdpi.com/article/10.3390/plants11243437/s1, Figure S1: TEM and light micrographs of transverse sections of *Magnolia grandiflora* young leaves.
