# Supplementary material for: Double Puzzle: Morphogenesis of the Bi-Layered Leaf Adaxial Epidermis of Magnolia grandiflora
Source: Plants (Basel). 2022 Dec 9;11(24):3437. doi: 10.3390/plants11243437 (PMC9785140; doi:10.3390/plants11243437)
Supplement: Supplementary file 1 [file plants-11-03437-s001.zip › plants-2010147-supplementary.pdf]

Double puzzle: Morphogenesis of the bi-layered leaf adaxial epidermis of *Magnolia grandiflora*

Emmanuel Panteris, Ioannis-Dimosthenis S. Adamakis

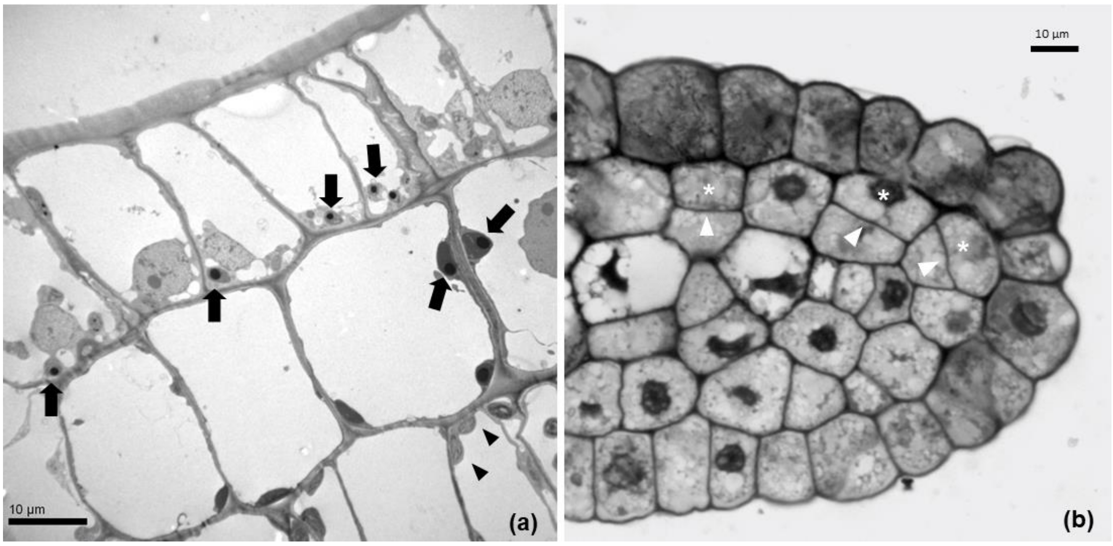

Figure S1. TEM (a) and light (b) micrographs of transverse sections of *Magnolia grandiflora* young leaves. (a) The cells of the external adaxial epidermis, as well as those of the hypodermis, contain plastids with globular electron-dense inclusions (arrows), unlike the chloroplasts (arrowheads) of underlying mesophyll cell layer. (b) Marginal area of very young leaf. Arrowheads point to periclinal walls in ground meristem cells just beneath the protoderm, separating the cells of the hypodermis (asterisks) from the rest ground tissue. Note that this separation occurs only under the adaxial protoderm, to produce the bi-layered epidermis. Scale bars: 10 μm.
